# The Royal College of Psychiatrists’ Leadership and Management Fellow Scheme

**DOI:** 10.1192/bjb.2021.31

**Published:** 2022-06

**Authors:** Alex Till, Deepa Bagepalli Krishnan, Russell Gibson, Michael Hobkirk, David Somerfield, Helen Crimlisk

**Affiliations:** 1 Health Education England (North West), UK; 2 Health Education England (East Midlands), UK; 3 Health Education England (Peninsula), UK; 4 Sussex Partnership NHS Foundation Trust, UK; 5 Devon Partnership NHS Trust, UK; 6 Sheffield Health and Social Care NHS Foundation Trust, UK

**Keywords:** Education and training, leadership and management, leadership development, psychiatric leadership

## Abstract

The Royal College of Psychiatrists’ Leadership and Management Fellow Scheme aims to develop and support a new cohort of leaders within psychiatry. This article provides an introduction to the scheme, which is accessible to all higher trainees with the support of their host organisation. We explore its development, structure and how it is evolving to provide a strong platform for achieving the College's ambition to benefit patient care by embedding a culture of medical leadership within mental health services.

The Royal College of Psychiatrists’ (RCPsych) Leadership and Management Fellow Scheme has been developed by the College's Psychiatric Trainees’ Committee (PTC), the Leadership and Management Committee (LMC) and the Centre for Advanced Learning and Conferences (CALC).

It is designed to support the formation of a national network of emerging medical leaders within psychiatry. It builds on the General Medical Council's Generic Professional Capabilities framework^1^ and, in accordance with the Faculty of Medical Leadership and Management's leadership and management standards for medical professionals,^2^ it provides an enhanced understanding of medical leadership at an individual, team, organisational and systemic level.

The College has committed to ‘provide high quality face-to-face training in leadership and management’ that specifically meets the needs of ‘psychiatrists making the transition to consultant grade’.^3^ This scheme, despite the necessity to adapt to an alternative delivery model as a result of the SARS-CoV-2 pandemic, fulfils that commitment. It realises the vision of both the LMC, ‘to develop and support psychiatrists as leaders and managers’,^4^ and of the PTC, to support trainees in having ‘greater autonomy over their careers through in-programme developmental opportunities’.^5^

It is critical that future doctors develop leadership values and behaviours as core attributes, and learn about leadership, followership and team working.^6^ For this to be successful, however, and for psychiatrists at every level to assume leadership responsibility, trainers and training organisations must ensure that leadership development is given parity with achieving clinical and academic competencies. It is only through this mechanism that we will truly achieve the notion of ‘expert leadership’^7^ within psychiatry.

This paper supplements Till *et al*^8^ which explores the importance of leadership, leadership development theory and opportunities available for psychiatric leadership development.^8^ We focus specifically on the development and structure of the RCPsych Leadership and Management Fellow Scheme as a mechanism to develop emerging medical leaders within psychiatry.

## Evidence in practice

The evidence for leadership and leadership development in healthcare is clear.^9^ An engaged medical workforce with capable, high-quality leaders has consistently been demonstrated to be integral to driving improvements and supporting high-quality care.^10,11^

Complementary to this, there is a growing call at a national level for services funded by the National Health Service (NHS) to strengthen their approach to leadership development and talent management,^12^ with future doctors ‘developing an appreciation of leadership at an early stage’.^6^

Leadership development, however, does not suit a ‘one size fits all’ approach. There is little robust evidence for the effectiveness of specific leadership development schemes and difficulty in evaluating their effectiveness.^9^ As a result, multiple leadership development programmes have emerged at a national, regional and local level, with various approaches undertaken to meet the diverse needs of doctors, depending on their stage of training, specialty and location.^8^

Traditionally, leadership development programmes have focused on so-called ‘horizontal’ leadership development, where individuals attend courses run by external providers which focus at an individual level on ‘what leaders need to know’: developing their knowledge, skills and competencies.^13^

Individual interventions alone, however, are insufficient.^14^ Developing leadership crucially depends on context, and leadership development must take place in the context in which an individual works. Within healthcare, this should develop organisational allegiance and engagement in the quality and safety agenda of organisations, maintain a systems focus to meet the challenges of increasingly integrated health and social care services, and develop a strong network between tomorrow's leaders.^15^ Furthermore, supported by a recent systemic review,^16^ mixed external and internal faculty is crucial, with project work and mentoring increasing the likelihood of good organisational outcomes.

The RCPsych Leadership and Management Fellow Scheme draws lessons from this knowledge, other schemes, and the latest educational leadership theory, to combine a national training programme with a local apprenticeship model, so that fellows not only experience ‘horizontal’ leadership development but are also exposed to ‘vertical’ leadership development.^13^

‘Vertical’ leadership development focuses on ‘how leaders think’, advancing interdependent styles of more complex, systemic and strategic thought.^13^ It relies on three primary conditions: ‘heat experiences’, ‘colliding perspectives’ and ‘elevated sense-making’.^17^ These are described further below, with illustrative quotations reflecting how fellows experienced these ways some of the scheme's fellows have experienced these are reflected in their illustrative quotations below. Fellows consented to anonymised quotes from evaluation data being included in publications.

Heat experiences place fellows in challenging situations where there is a degree of risk. Fellows experience this both in the classroom and through the leadership projects undertaken in their place of work. This is the ‘what’ that disrupts and disorients their traditional thinking to discover new and better ways to make sense of the challenges they face as they assume increasing leadership responsibility:

‘The service development project consolidated our more abstract learning. It helped me get first-hand experience of the challenges faced being a leader, how I might need to develop and adapt my leadership style in different situations, and to become more self-aware and collaborative in my approach to leadership’.

Colliding perspectives, on the other hand, are the ‘who’. Fellows are exposed to leaders both across the system and within their local organisations, including patient and carer leaders. When combined with the community of learning that they establish with their peers from around the country, fellows are enabled to gather different views and opinions, challenge their existing mental models^18^ and see the world through diverse new perspectives:

‘I valued the opportunity to work with different trainees across the country, hear from eminent leaders about their leadership journeys, and meet individuals within the management hierarchy of the organisation. It allowed us to share experiences and learn from each other, to de-mystify senior leadership roles, and to connect and gain better understanding of those in managerial roles’.

Elevated sense-making is the final piece, the ‘how’. In combination with their ‘consulting pairs’, where fellows are partnered to coach each other through the scheme, and senior mentoring locally, fellows are afforded the space to reflect and begin integrating their experiences and new perspectives, to advance their action logics^19^ and leadership effectiveness:

‘The opportunity to reflect and role-play was very useful and very powerful. It enabled me to see the consequences of my behaviour and how it might affect others. My experience of working with my organisation mentor was also very positive, she really helped me work though fervent leadership dilemmas’.

## Development

The RCPsych Leadership and Management Fellow Scheme was founded through a joint venture between the College's Psychiatric Trainees Committee, the Leadership and Management Committee and the Specialist Advisor for Workforce. It was first proposed to the Education and Training Committee in February 2017, and although originally modelled on the Royal College of Physicians’ (London) Chief Registrar Scheme, it adopted an alternative model and evolved to embrace a more inclusive approach.

To achieve this, the scheme was modified to be potentially accessible to all higher trainees in psychiatry, including those in less than full time (LTFT) training, without any extension to the duration of their training. It occurred ‘in-programme’, across a 12-month period, averaging 1 day per week, utilising trainees’ protected special interest time. This minimised provider costs, with no requisite for funding fellows' salaries or clinical backfill.

Medical directors from mental health providers across the UK were highly supportive of the scheme, and in the absence of central funding, were prepared to make a financial investment of £2000 per trainee, with full or partial self-funding (including via the trainee's study budget) excluded to maximise equity of access and ensure organisational commitment.

With this early adoption, a competitive tendering process was pursued, with the RCPsych Centre for Advanced Learning and Conferences (CALC) being selected above highly competitive offers from multiple business schools and the Faculty of Medical Leadership and Management (FMLM) to deliver the national leadership development training programme.

Recruitment was delegated to host organisations and supported by training-programme directors, with statements required that fellows had been identified and nominated through an open and competitive process, as determined locally.

We welcomed the first cohort of RCPsych Leadership and Management Fellows in September 2019, with an initial intake of 30 fellows from 19 different service providers; over half were female (57%) and from Black and minority ethnic communities (63%).

## Structure

The RCPsych Leadership and Management Fellow Scheme is designed to support the formation of a national network of emerging medical leaders within psychiatry and develop their confidence and ability to operate within and lead across a range of mental health organisations and systems to improve patient care.

It combines a bespoke, high-impact leadership development training programme with a local apprenticeship model, where fellows are mentored by senior medical leaders within their organisations and proactively engage in a variety of leadership projects.

### National training programme

Building on the College's extensive experience in providing highly regarded leadership and management training, the evidence-based bespoke leadership development training programme is facilitated by the programme faculty, in conjunction with outside expertise where relevant. It is designed to enhance a range of practical knowledge and skills that consider leadership from the perspective of four behavioural domains relating to self, team, organisation and system, as outlined by the FMLM's Leadership and Management Standards for Medical Professionals.^2^ A detailed up-to-date programme is available from the College on request.

Although originally designed for face-to-face learning, the implications of the SARS-CoV-2 pandemic were felt with our first cohort, and we took this as an opportunity to adopt an exciting new digitally focused approach, with the same content delivered virtually.

A central focus of the programme is to develop an underpinning community of learning, as fellows are afforded the space to reflect on their own leadership style, and conditions are established in which collaborative relationships can be optimised as they network with peers, share their experiences and learn collaboratively from best practice across the multiple organisations they represent.

Fellows are additionally uniquely exposed to inspirational national leaders from across the system, with guest speakers, including a number of prominent psychiatric leaders with national roles both within and outside the College, integrated throughout the programme.

### Local mentoring and support

Fellows are supported and mentored throughout the scheme by a senior medical leader within their organisation, who commits to a minimum of six mentoring sessions throughout the 12-month duration of the scheme when nominating a fellow. This is a key aspect of the scheme and is essential to the success of the fellow's role.

The expectation is for the mentoring role to be held by the medical director or a nominated deputy of sufficient seniority and, where appropriate, individual project supervision may be delegated to a clinical director.

In conjunction with this, fellows should also have opportunities to shadow at an executive level and with key external health and local authority partners. This aims to generate a deeper understanding of healthcare leadership and management within the wider social, political and economic context.

### Leadership projects

A fundamental component of the scheme is the development of fellows as apprentice leaders through their engagement in a variety of leadership projects within their local organisations.

The exact nature of leadership projects is negotiated and managed locally between the fellow and their mentor. Fellows can join existing larger projects or develop their own smaller projects, although it is stipulated that they should be guided to ensure that all projects are of strategic or operational significance to the organisation, ensuring that fellows make a meaningful contribution and organisations gain a return on their investment. Some examples of leadership projects are given in Box 1.

Box 1 Examples of leadership projects undertaken by RCPsych Leadership and Management Fellows 2019–2020

- Trust-wide leadership and training in quality improvement
- Quality improvement projects related to high-dose antipsychotic prescribing, reducing restrictive practices under the Mental Health Act, and the co-production of service developments to amplify the patient voice and improve patient experience
- Trust-wide policy developments related to the management of dual diagnosis, COVID-19, video consultations, physical health management and electrocardiogram (ECG) monitoring
- Pathway developments related to naloxone prescribing in general hospitals, management of medically unexplained symptoms, attention-deficit hyperactivity disorder (ADHD) in child and adolescent mental health services, and clinical decision units within forensic services

Recognising that projects can evolve, emerge and falter for various reasons, not least a worldwide pandemic, successful completion of the RCPsych Leadership and Management Fellow Scheme is not conditional on the ‘success’ of a fellow's project. There is an explicit recognition that learning can occur irrespective of this, and that a fellow's success is rather more meaningfully determined by their engagement with the programme, their reflective practice and their mentor's feedback, with their learning, growth and leadership development assessed throughout the scheme.

## Benefits

Boxes 2 and 3 summarise two fellows' experiences of participating in the scheme, with Box 4 outlining the intended benefits of the RCPsych Leadership and Management Fellow Scheme for individual fellows, the organisations in which they work, and for patient care.

Box 2 Vignette 1: a fellow's experience of the RCPsych Leadership and Management Fellow Scheme

‘I feel this fellowship has provided me with the foundations to further acquire the knowledge and skills that are relevant in the context of leading a complex healthcare system. The opportunity to lead a trust-wide project with the support of my mentor allowed me to work collaboratively with multidisciplinary professionals across organisations and think about change management and sustainability in a very different way. Combined with the deeper insight into leadership theories I gained through the national training programme, and the reflective nature of the sessions, which I particularly enjoyed, I now feel more confident in leading service improvement projects and in engaging with diverse stakeholder groups. Furthermore, I found that being part of a national scheme, whilst being supported locally by a senior mentor, helped me build links and network with peers and senior leaders both locally and nationally. This was a unique feature of the scheme and I have no doubt it will help me in my future role as a consultant in the organisation.’

Box 3 Vignette 2: a fellow's experience of the RCPsych Leadership and Management Fellow Scheme

‘One of the great advantages of the scheme was the direct support from a senior mentor to get “hands on” in a significant trust-wide project. Like many other trainees, I had previously been involved in small-scale projects, but had never been given responsibility for developing such a complex intervention across multiple community teams. Whilst simultaneously daunting and exciting, I found the direct support of my mentor invaluable in negotiating the complexity of the trust systems, while working collaboratively with a number of colleagues from different backgrounds, including project management, IT and senior managers, in addition to key clinical staff who would be delivering the intervention. Throughout the scheme my mentor was able to help me keep on track with the project and helped me to identify and keep in mind the vision of we wanted to achieve. I now feel much more able to lead change, effectively advocate for improved patient care, and am better prepared for the challenges of starting as a consultant.’

Box 4 Intended benefits of the RCPsych Leadership and Management Fellow Scheme

**Benefits for patients and the organisation**

- High-quality care: increase the number of highly skilled medical leaders able to develop and foster collaborative practice and high-quality care
- Improved services: bring an enthusiastic and fresh perspective with committed time to help improve the safety and quality of your services and help create a culture of continuous improvement
- Enhanced medical engagement: fellows hold mutually enhancing conversations with trainees, senior leaders and management to boost the performance of the organisation
- Emerging medical leaders: invest in your local talent and nurture the next cohort of medical leaders within your organisation

**Benefits for the individual**

- Heightened self-awareness: gain a deeper understanding of which aspects of yourself enable or constrain your leadership
- Discover your inner leader: begin or continue the process of discovering and releasing your leadership potential
- Mentoring, networking and support: learn from senior medical leaders and develop an inspiring network of like-minded peers, including from within the RCPsych Leadership and Management Fellow Scheme Alumni Network, which fellows will be invited to join on completion of the scheme
- Leadership and management skills: develop your understanding and a widening repertoire of leadership competencies and skills that will help you be a better medical leader and apply for associate fellowship of the Faculty of Medical Leadership and Management
- Followership skills: gain a greater understanding of the role that followers play in the co-construction of leadership identities
- Career autonomy: take control of your career and increase your confidence in extending your portfolio and building a local and national profile

## Evolution

The RCPsych Leadership and Management Fellow Scheme is an evolutionary process and key to its future development will be feedback from both fellows and mentors, the needs of our healthcare system and the sociocultural needs of the wider society we lead within.

Although the College has a well-established reputation for delivering leadership and management training, this is the first developmental scheme for trainees. It emerged organically, being co-produced by those for whom it was intended. The scheme will continue to be developed on this basis in order to further adapt and respond to the specific needs of higher trainees in psychiatry, provide value to the sponsoring organisations and achieve FMLM accreditation.

We are proud of our first cohort in 2019–2020, who despite the SARS-CoV-2 pandemic all successfully completed the scheme. We are also pleased to have been able to adapt to an exciting new digitally focused approach for 2020–2021, allowing for more regular contact that will be supported by a new online platform to promote networking, shared learning and reflection.

We acknowledge the challenges that the loss of face-to-face learning involves, particularly regarding the development of close and trusting relationships, but believe that a digital approach will be critical for the future. It not only ensures that mental health services are capable of weathering the current storm, but also that leadership capabilities within the psychiatric workforce are still developed and able to rise to future challenges as we deal with the mental health implications of the SARS-CoV-2 pandemic alongside the implementation of the NHS Long Term Plan.^20^

Reassuringly, however, although we and many others look forward to the return of face-to-face learning, research from the Centre for Creative Leadership^21^ has highlighted similar levels of results for online leadership training, while providing the additional benefits of increased convenience and accessibility.

Combined with increasing confidence and familiarity with digital engagement, we will therefore integrate these opportunities as face-to-face learning returns and embrace a more blended approach. We hope that this increased flexibility will widen access to the scheme for Category 1 less than full time (LTFT) trainees (i.e. those with health reasons or caring responsibilities) and for those living at a greater geographical distance from the College, by reducing face-to-face learning.

Providing additional value, current alumni have been invited to facilitate action learning sets, and to join the programme faculty for future cohorts. They have also established an RCPsych Leadership and Management Fellow Scheme Alumni Network. This will be integrated with the RCPsych Leadership and Management Committee and future cohorts of the RCPsych Leadership and Management Fellow Scheme. It aims to maintain links between fellows as they become future leaders within mental healthcare across the UK, nurture the next generation through near-peer mentoring, promote collaboration across the system, develop shared learning and good practice, and strengthen the connection between mental health services and the College.

The success of this scheme highlights the appetite for strong medical leadership among progressive healthcare organisations throughout the UK, who recognise the importance of advanced leadership development schemes for aspiring organisational and system leaders.

The scheme has also unearthed a wider unmet need for leadership development among specialty doctors, new consultants and other groups; they of course have their own unique challenges that deserve appropriate recognition. Appreciating this, the scheme is diversifying and strengthening its leadership faculty in order to improve its offer, strengthen its resilience and develop greater resources from which to further develop the leadership and management skills of the wider membership.

## Conclusions

Leadership and management is for all doctors, for all psychiatrists at every level. It is not just for those with formal roles within organisational hierarchies who hold designated leadership positions. Nor is it about stand-alone heroic individuals: it collectively lives among us as a professional body and we must learn to nurture, support and value one another.

The RCPsych Leadership and Management Fellow Scheme is just one step towards developing leaders in psychiatry. Through the combination of a national training programme with a local apprenticeship model, both formal and informal leadership development is provided, where emerging leaders have a safe space to take risks, experiment and develop ‘on the job’.

It is important to recognise, however, that leadership development does not suit a ‘one size fits all’ approach. Whether through this scheme or another, we each have a responsibility to collectively develop and establish a culture that nurtures leadership talent and ultimately improves the lives of people with mental illness.

## Data Availability

The data that support the findings of this study are availablefrom the corresponding author, A.T., upon reasonable request.

## About the authors

**Alex Till**, MBChB, MRCPsych, MSc, MBA, is a specialty registrar in forensic psychiatry with Health Education England (North West), UK, and was Chair of the Royal College of Psychiatrists’ Psychiatric Trainees Committee in 2018–2019. **Deepa Bagepalli Krishnan**, MBBS, MRCPsych, PGDip, Dip IBLM/BSLM, is a Clinical Assistant Professor in the Faculty of Medicine and Health Sciences, University of Nottingham, UK, and a speciality registrar in general adult psychiatry with Health Education England (East Midlands); she was an RCPsych Leadership and Management Fellow in 2019–2020. **Russell Gibson**, BSc(Hons), MBBCh, MRCPsych, MSc, is a specialty registrar in general adult and old age psychiatry with Health Education England (Peninsula), UK, and was an RCPsych Leadership and Management Fellow in 2019–2020. **Michael Hobkirk**, FRCPsych, MSc, MMedEd, FHEA, is a consultant child psychiatrist and Director of Medical Education with Sussex Partnership NHS Foundation Trust, and former College Lead for Training, Royal College of Psychiatrists, UK. **David Somerfield**, FRCPsych, is Medical Director and a consultant psychiatrist with Devon Partnership NHS Trust, UK. **Helen Crimlisk**, FRCPsych, MSc (Ashridge), FAcadMEd, is Deputy Medical Director of Sheffield Health and Social Care NHS Foundation Trust and an Associate Registrar for Leadership and Management, Royal College of Psychiatrists, UK.
